# Supplementary material for: Little-Parks like oscillations in lightly doped cuprate superconductors
Source: Nat Commun. 2022 Mar 14;13:1316. doi: 10.1038/s41467-022-28954-w (PMC8921203; doi:10.1038/s41467-022-28954-w)
Supplement: Supplementary file 1 — Supplementary information [file 41467_2022_28954_MOESM1_ESM.pdf]

Supplementary information to:

Little-Parks like oscillations in lightly doped cuprate superconductors

Menghan Liao[1], Yuying Zhu[2], Shuxu Hu[1], Ruidan Zhong[3], John Schneeloch[3,4], Genda Gu[3], Ding Zhang [1,2,5,6*], and Qi-Kun Xue[1,2,5,7*]

[1] *State Key Laboratory of Low Dimensional Quantum Physics and Department of Physics, Tsinghua University, Beijing 100084, China*

[2] *Beijing Academy of Quantum Information Sciences, Beijing 100193, China*

[3] *Condensed Matter Physics and Materials Science Department, Brookhaven National Laboratory, Upton, New York 11973, USA*

[4] *Department of Physics and Astronomy, Stony Brook University, Stony Brook, New York 11794, USA*

[5] *Frontier Science Center for Quantum Information, Beijing 100084, China*

[6] *RIKEN Center for Emergent Matter Science (CEMS), Wako, Saitama 351-0198, Japan*

[7] *Southern University of Science and Technology, Shenzhen 518055, China*

*Corresponding author.

Email: dingzhang@mail.tsinghua.edu.cn, qkxue@mail.tsinghua.edu.cn

Contents

Supplementary Note 1. Estimation of B_{c2} and ξ_0 in under-doped Bi-2212

Supplementary Note 2. Resistance oscillations in under-doped Bi-2212 on SiO_2/Si substrates

Supplementary Note 3. Resistance oscillations in a Bi-2212 microbridge

Supplementary Note 4. Exclusion of extrinsic mechanisms for the low field resistance oscillations

Supplementary Note 5. Subtraction of the smoothed background

Supplementary Note 6. Doping dependence of the extracted fitting parameters

Supplementary Note 1. Estimation of B_{c2} and ξ_0 in under-doped Bi-2212

The measurement of B_{c2} in cuprate superconductors is not as straightforward as low-temperature superconductors. Not only B_{c2} often exceeds the highest available magnetic field, but also there exists complications brought by the vortex solid and liquid phases [1-4]. Previous studies often rely on measuring the Nernst effect instead of simple resistance measurements to determine B_{c2}. However, measurements of B_{c2} in the extremely underdoped regime ($p \leq 0.1$) remain scarce.

Based on our transport data, we obtain the magnetic field at which the vortex solid melts—B_{vs} at different temperatures below T_{c0}. Although $B_{vs}(T)$ is usually lower than the upper critical field $B_{c2}(T)$ at finite temperatures, previous experiments on a variety of cuprate superconductors (overdoped Tl-Bi2201, underdoped YBCO [2, 3], $Nd_{2-x}Ce_xCuO_4$ [1]) showed that $B_{vs}(T)$ becomes very close or even merges with $B_{c2}(T)$ at $T \rightarrow 0$ K. We therefore extrapolate B_{vs} down to 0 K to derive a lower limit of B_{c2}. Based on $B_{c2}(0)$, one can readily calculate the values of ξ_0 by using the formula: $\xi_0 = \sqrt{\frac{\Phi_0}{2\pi B_{c2}}}$.

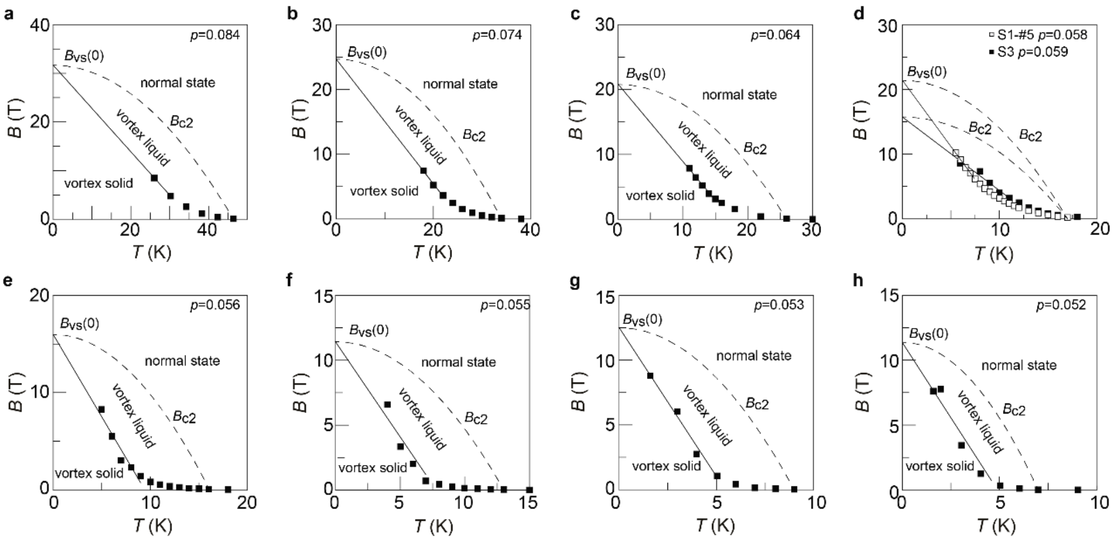

Supplementary Figure 1. Magnetic-field and temperature phase diagrams of underdoped Bi-2212. Data points indicate B_{vs} at a set of fixed T. They mark the magnetic field at which the magneto-resistance reaches 10% of the zero-field resistance value at 87 K. Filled symbols are for sample S3 at different doping levels. Empty symbols in **d** are extracted from sample S1 at the fifth gated stage. Solid lines are the linear extrapolations based on the data points at low temperatures. Dashed curves are a guide to the eye, showing the expected temperature evolution of the upper critical field B_{c2} [3].

Supplementary Figure 1 shows the temperature dependence of B_{vs} for two samples discussed in the main text and at different doping levels. Here we define B_{vs}

as the field where the magneto-resistance reaches 10% of the normal state resistance (zero-field resistance at 87 K). Although a theoretical formula describing $B_{vs}(T)$ can be employed for fitting the data at finite temperatures [2], we find that this fitting gives rise to a large uncertainty of $B_{vs}(T \to 0)$ at small p. This uncertainty is mainly caused by the limited portion of the temperature window below T_{c0} that can be covered experimentally. Instead, we carry out a simpler evaluation by linearly extrapolating the data points to absolute zero (Solid lines in Supplementary Figure 1). The extrapolated values of $B_{vs}(0)$ are then taken as equal to $B_{c2}(0)$.

In Supplementary Figure 2, we plot the estimated $B_{c2}(0)$ as well as the calculated ξ_0 as a function of the doping level. The decreasing trend of B_{c2} as p decreases can also be inferred from the reduced B_{vs} at lower p in Supplementary Figure 1. Previous studies only measured Bi-2212 at $p \geq 0.1$ such that a direct comparison to our estimated ξ_0 at $p < 0.1$ is not possible. Nevertheless, we include in Supplementary Figure 1 the reported $B_{c2}$and ξ_0 values for YBCO at $p < 0.1$. They show consistent values and doping dependence as those of our estimated values in Bi-2212.

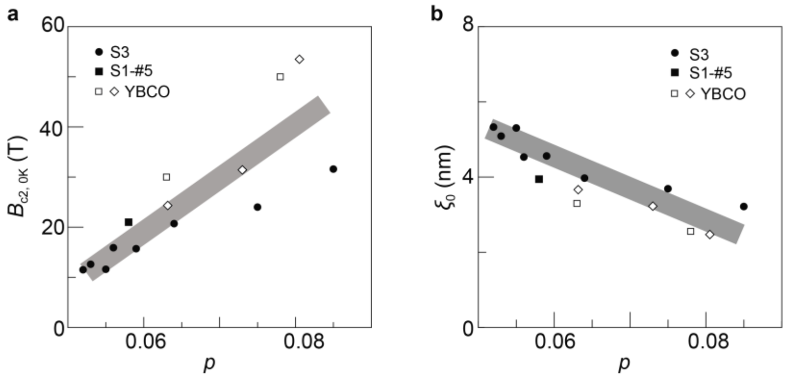

Supplementary Figure 2. Estimated B_{c2} and ξ_0 in underdoped Bi-2212. a, Filled symbols show the doping dependence of B_{c2} of underdoped Bi-2212 obtained from the linear extrapolations in Supplementary Figure 1. Empty diamonds and squares are the B_{c2} values of YBCO from ref. [2] and [3], respectively. **b**, Calculated ξ_0 of Bi-2212 and YBCO based on the corresponding B_{c2} in panel **a**.

Supplementary Note 2. Resistance oscillations in under-doped Bi-2212 on SiO_2/Si substrates

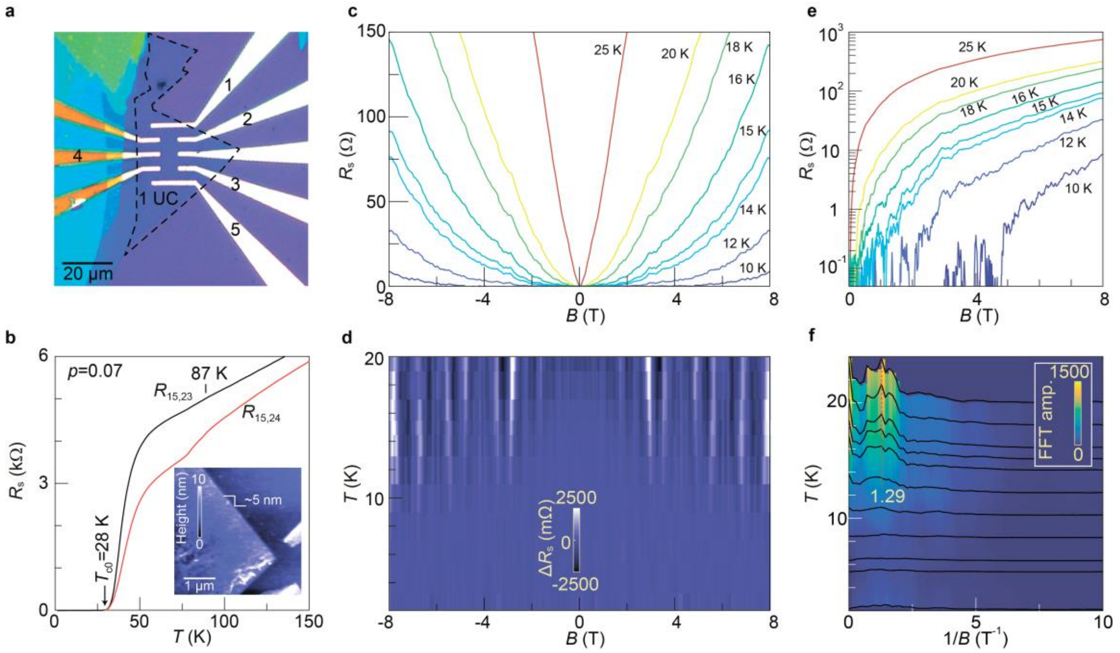

Supplementary Figure 3. Magneto-transport results of an ultrathin Bi-2212 flake (1 UC) on the SiO_2/Si substrate. a, Optical image of sample S5. The dashed line demarcates the 1 UC region. Specific contacts used in transport are marked with numbers. **b**, Sheet resistance of S5 as a function of temperature. Red (Black) curve is obtained by passing the current from contact 1 to contact 5, as indicated in **a**, and measuring the voltage between contacts 2 and 4 (2 and 3). The kink at around 87 K is presumably from the thicker flakes contacted by electrode-4 (see panel **a**), which remain in the initial doping regime. Inset shows the AFM image of S5 after the transport measurements. The step height is about 5 nm. **c**, Sheet resistance of S5 as a function of magnetic field at a set of temperature points. **d**, Color plot of the background subtracted sheet resistance of S5. **e**, Same magneto-resistance traces as in **c** but plotted with the *y*-axis in log scale to enhance the oscillatory features. **f,** FFT of the background subtracted resistance traces at different temperatures. Vertical dotted line with the number marks the peak positions.

We observe similar resistance oscillations in exfoliated Bi-2212 flakes on SiO_2/Si substrates. These samples are not intercalated by lithium ions. They are driven into the under-doped regime by varying the oxygen content directly. Supplementary Figure 3 shows the data of one such sample—S5. The thickness is about 1 UC, as can be roughly determined by the optical contrast (Supplementary Figure 3a). The sample was further capped by a thin flake of hexagonal boron nitride (hBN) for protection against air exposure.

Supplementary Figure 3b shows the temperature dependent resistance of S5. The resistance values are similar to that reported previously in 1 UC Bi-2212 [4], reaffirming

the ultrathin nature of our flake. Clearly, S5 possesses a T_c of 28 K, indicating that it is in the underdoped regime. The reduction of doping is caused by natural oxygen loss during the sample processing in the glovebox. Similar loss of oxygen for the ultrathin Bi-2212 flake was reported before [5].

Supplementary Figure 3c illustrates the magneto-resistance at different temperatures. Oscillatory features start to appear symmetrically around $B = 0$ T below 25 K. In Supplementary Figure 3d, we show the color-coded data with the smoothed background subtracted. Prominent zebra stripes occur around the superconducting transition region. In Supplementary Figure 3e, the magneto-resistance is shown in a logarithmic scale. Both plots (panels d and e) show repeatable oscillations at various temperatures. Furthermore, the FFT of the background subtracted traces (Supplementary Figure 3f) gives rise to a peak at around 1.29 T^{-1}, corresponding to ΔB of about 0.78 T. This period is similar to that obtained in lithium intercalated Bi-2212.

After the transport measurements, we peeled off the hBN layer and measured the sample thickness by atomic force microscopy (AFM). The AFM image is shown as an inset of Supplementary Figure 3b. The step height at the edge of the sample is about 5 nm. This apparent thickness of the Bi-2212 flake is thicker than the nominal value of 3 nm for 1 UC flake. As found previously, the thickness determined by AFM is usually larger than the theoretical values, presumably due to the different chemical potentials of Bi-2212 and SiO_2 [4].

A second Bi-2212 flake on SiO_2/Si, which shows low-field oscillations, is presented in the next section.

Supplementary Note 3. Resistance oscillations in a Bi-2212 microbridge

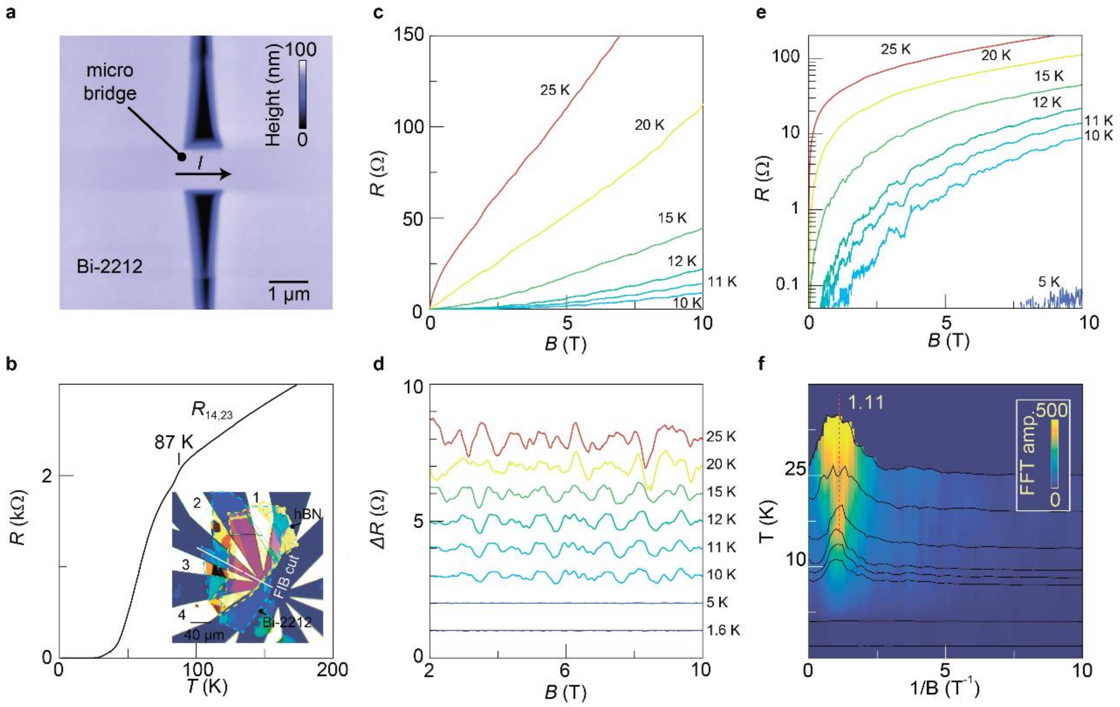

Supplementary Figure 4. Resistance oscillations of a Bi-2212 micro-bridge. a, AFM image of the Bi-2212 micro-bridge. **b**, Temperature dependent resistance of the sample. The kink at 87 K can be attributed to the region farther from the micro-bridge, which remains the initial doping level. Inset shows an optical image of the device. The central part of the Bi-2212 flake (marked by blue dashed lines) is covered by h-BN (yellow dashed line) for protection. White straight line indicates the cutting direction for the focused ion beam (FIB). The resistance is measured by passing the current from contact 1 to 4 and measuring the voltage between contacts 2 and 3. **c**, Magneto-resistance traces at a set of temperatures. **d**, Background subtracted magneto-resistance at different temperatures. **e**, Same magneto-resistance traces as in **c** but plotted with the *y*-axis in log scale to enhance the oscillatory features. **f**, FFT of the background subtracted resistance traces at different temperatures. Vertical dotted line with the number marks the peak positions.

To investigate the mesoscopic effect as a possible cause for the low-field oscillations, we intentionally pattern a microbridge in Bi-2212 flake. Supplementary Figure 4a and inset of Supplementary Figure 4b display our sample. The Bi-2212 flake is about 20 nm thick (~6 UC) and is capped by hBN for protection. A microbridge with a width of 1 μm was patterned by using focused ion beam (FIB) with Ga^+ ions at 15 kV. The FIB cutting direction is schematically shown in the optical image in the inset of Supplementary Figure 4b.

Supplementary Figure 4b shows the temperature dependent resistance, indicating that $T_{c0} = 25$ K. It suggests that the sample is in the underdoped regime with $p =$ 0.067. Again, the flake was exfoliated from the nearly optimal doped bulk crystal. It

became underdoped presumably due to the oxygen loss during the FIB process.

Supplementary Figure 4c-e show the magneto-resistance traces and the background subtracted ones. The oscillation amplitude is comparable to those observed in flakes without patterning the micro-bridge. Furthermore, the FFT in Supplementary Figure 4f shows a peak at 1.11 T^{-1}, which is consistent with the values obtained from other samples. These results suggest that the low-field oscillations show no apparent dependence on the channel width in the range from 1 μm to 80 μm. The mesoscopic effect seems to play a negligible role in giving rise to the oscillations.

Supplementary Note 4. Exclusion of extrinsic mechanisms for the low field resistance oscillations

Supplementary Figure 5 shows the resistance curves obtained at the same temperature but with different field-sweep directions or sweep rates. We also measured the resistance with different currents. All of the curves show oscillating features at the same magnetic field. External perturbation such as electrical noise, flux jump in the superconducting magnet, temperature instability, etc. can be therefore excluded as the reason for the oscillations.

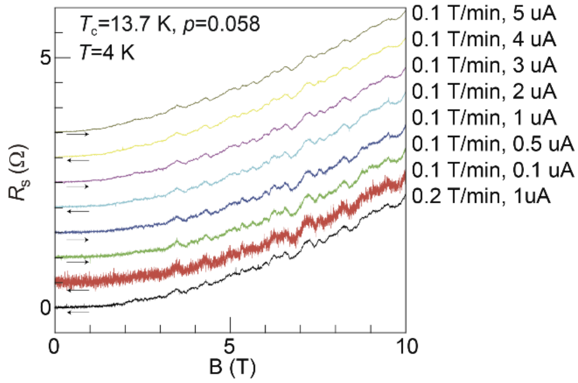

Supplementary Figure 5. Magneto-resistance of sample S1. Curves are obtained with different excitation currents, different sweep rates and directions of magnetic field. The sweep directions are marked by the black arrows. After obtaining the data for the bottom most curve, we did thermal cycling up to 120 K and back to 4 K. The rest of the curves were obtained from the second cool-down.

Supplementary Note 5. Subtraction of the smoothed background

To obtain the data shown in Figs. 1d, 2b, S3d and Supplementary Figure 4d, we subtract the original magneto-resistance by smoothed backgrounds. To obtain the smoothed backgrounds, we take the following steps. First, we interpolate the measured data such that the magnetic field spacing is 1 mT, i.e. 1000 points/T. Then, we run a moving average over 1000 data points to obtain the background curve. In Supplementary Figure S6, we compare the data obtained by subtracting such a background to that from a different method. In Supplementary Figure 6a**,** the subtracted background is still the moving averaged one. To obtain the data shown in Supplementary Figure 6b, we subtract a background that is a polynomial fitting to the original magneto-resistance (to the seventh order). The right panels of Supplementary Figure 6 compare the FFT results of the data obtained by the two methods. In general, they show qualitatively no difference and the oscillation periods obtained are the same.

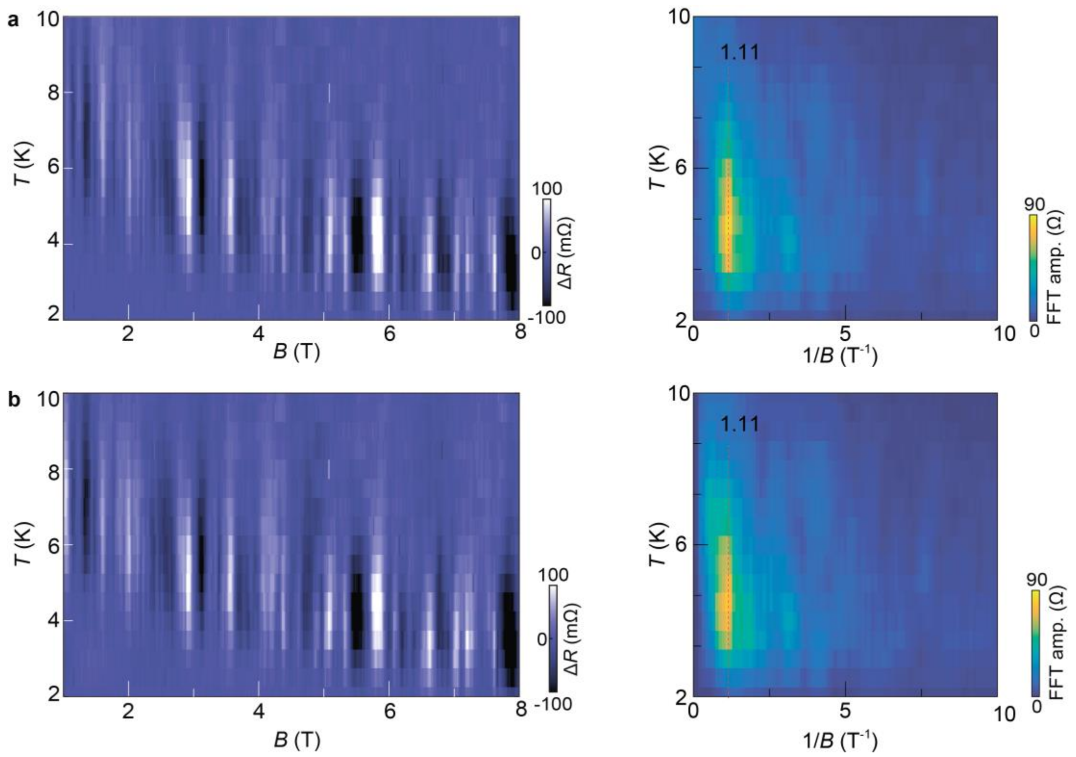

Supplementary Figure 6. Comparison of the resistance oscillations obtained by two different background subtraction methods. a, (Left) ΔR as a function of magnetic field and temperature. (Right) FFT of $\Delta R(B)$ at different T. Here, the subtracted background is obtained by smoothing the original curve in a range of 1 T (running average). **b**, ΔR and its FFT of the same data set of magneto-resistance. However, ΔR is obtained by subtracting a polynomial fit to the original curve.

Supplementary Note 6. Doping dependence of the extracted fitting parameter

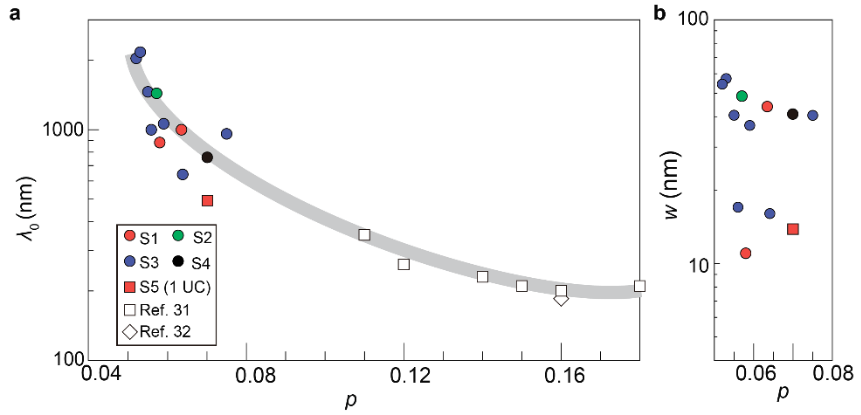

Supplementary Figure 7. Doping dependence of the extracted fitting parameter $\boldsymbol{\lambda_0}$ and $\boldsymbol{w}$. Filled symbols in A and B are the data points from our fitting. Empty squares in A are measured penetration depth in Bi-2212 [6]. Empty diamond in A is the reported value for Bi-2212 in ref. [7].

p	ξ_0 (nm)	a (nm)	T_c (K)	R_n (Ω)	w (nm)	λ (nm)
0.074	3.68	42.1	35	0.14	40.8	960
0.064	3.97	52.4	30	0.71	16	640
0.059	4.56	38.5	21	0.63	37	1060
0.056	4.53	45.5	20	2.5	17	1000
0.055	5.3	42.2	18	1.42	41	1460
0.053	5.1	57.0	15	4.83	57	2220
0.052	5.3	54.4	15	4	54	2040

Supplementary Table 1. Summary of parameters for sample S3 at different doping levels. Here the doping level p is evaluated by using the experimentally measured T_{c0} and the formula: $T_{c0} = T_{c,max}[1 - 82.6(p - 0.16)^2]$. The coherence length ξ_0 is estimated from the magneto-transport data, as explained in supplementary information, section 1. a is the period extracted from the resistance oscillations. The rest of the parameters: T_c (critical temperature), R_n (normal state resistance), w (separation of the non-superconducting region in the superconducting mesh, as illustrated in Fig. 4a) and λ (penetration depth) are used in Eq. (1). In addition, the superconducting thickness in Eq. (1) is chosen to be: $d = 1.5$ nm, which is the thickness of half of a unit cell of Bi-2212. We choose this value because Bi-2212 is a layered superconductor with strong anisotropy and weak interlayer coupling.

References

[1] Y. Wang, et al. Dependence of upper critical field and pairing strength on doping in cuprates. *Science*, **299**, 86-89 (2003).

[2] J. Chang, et al. Decrease of upper critical field with underdoping in cuprate superconductors. *Nat. Phys.* **8**, 751-756 (2012).

[3] G. Grissonnanche G, et al. Direct measurement of the upper critical field in cuprate superconductors. *Nat. Commun*. **5**, 1-8 (2014).

[4] D. Jiang, et al. High-T_c superconductivity in ultrathin $Bi_2Sr_2CaCu_2O_{8+x}$ down to half-unit-cell thickness by protection with graphene. *Nat. Commun*. **5**, 1-8 (2014).

[5] Y. Yu, et al. High-temperature superconductivity in monolayer $Bi_2Sr_2CaCu_2O_{8+\delta}$ *Nature*, **575**, 156-163 (2019).

[6] X. Zhao, et al. Correlation between T_c and n_s/m^* in $Bi_2Sr_2CaCu_2O_{8+\delta}$ single crystals. *Physica C*, **307**, 265-270 (1998).

[7] M. Weber, et al. Magnetic-flux distribution and the magnetic penetration depth in superconducting polycrystalline $Bi_2Sr_2Ca_{1-x}Y_xCu_2O_{8+\delta}$ and $Bi_{2-x}Pb_xSr_2CaCu_2O_{8+\delta}$. *Phys. Rev. B.* **48,** 13022 (1993).
